# Depression, suicidal ideation, and associated factors: a cross-sectional study in rural Haiti

**DOI:** 10.1186/1471-244X-12-149

**Published:** 2012-09-19

**Authors:** Bradley H Wagenaar, Ashley K Hagaman, Bonnie N Kaiser, Kristen E McLean, Brandon A Kohrt

**Affiliations:** 1 Department of Epidemiology, Rollins School of Public Health, Emory University, Atlanta, GA, USA; 2 Hubert Department of Global Health, Rollins School of Public Health, Emory University, Atlanta, GA, USA; 3 Department of Anthropology, Emory University, Atlanta, GA, USA; 4 Department of Behavioral Sciences & Health Education, Rollins School of Public Health, Emory University, Atlanta, GA, USA; 5 Transcultural Psychosocial Organization (TPO) Nepal, Kathmandu, Nepal

## Abstract

**Background:**

Since the 2010 earthquake in Haiti, there has been increased international attention to mental health needs throughout the country. The present study represents one of the first epidemiologic studies of depression symptomatology, suicidal ideation, and associated factors in Haiti’s Central Plateau.

**Methods:**

We conducted a cross-sectional, zone-stratified household survey of 408 adults in Haiti’s Central Plateau. Depression symptomatology was assessed with a culturally-adapted Kreyòl version of the Beck Depression Inventory (BDI). Multivariable linear and logistic regression models were built using backward elimination, with the outcomes being continuous BDI scores and endorsing suicidal ideation, respectively.

**Results:**

The mean BDI score was 20.4 (95% confidence interval [CI]: 19.3-21.5), and 6.13% (N = 25) of participants endorsed current suicidal ideation. Factors associated with BDI scores were: continuous age (adjusted beta [aβ]: 0.14, CI: 0.06-0.22), female gender (aβ: 2.1, CI: 0.18-4.0), suicidal ideation (aβ: 11.1, CI: 7.3-14.9), death in family (aβ: 2.7, CI: 0.57-4.9), and prior life-threatening illness (aβ: 2.6, CI: 0.77-4.5). Education was a risk factor for depression among women but not among men, and employment was a risk factor for both genders. Factors associated with endorsing suicidal ideation were: BDI score (ten point change) (adjusted odds ratio [aOR]: 2.5, CI: 1.7-3.6), lack of care if sick (aOR: 5.5, CI: 1.1-28.6), alcohol use (aOR: 3.3, CI: 1.3-8.2), and ever having been to a Vodou priest (aOR: 3.2, CI: 1.1-9.5).

**Conclusions:**

A large proportion of Haiti’s Central Plateau may be experiencing high levels of depression symptomatology and/or current suicidal ideation. Screening could be conducted in biomedical, religious, and Vodou healing contexts. For prevention, poverty reduction and improved healthcare access are key elements. For treatment, general psychiatric services, psychosocial services for the medically ill and their families, and substance abuse interventions should be explored. Paradoxical associations related to education and employment require further exploration.

## Background

The earthquake in Haiti on January 12, 2010 led to a large response by international mental health and psychosocial workers [1]. While the psychological sequelae of the earthquake were the expected focus of attention, the event also highlighted chronic unmet mental health needs throughout the country [2,3]. In Haiti, little is currently known regarding the prevalence of, or risk factors associated with, depression and/or suicide. Addressing the knowledge gap of predictors for depression and suicide is critical given the anticipated burden of mental health disorders in Haiti, and in light of the 2010 earthquake that killed approximately 220,000 people and displaced 1.5 million more [4]. Although the population-level impact of natural disasters on depression and suicidal behavior varies significantly across studies in disparate settings [5-15], the World Health Organization (WHO) predicted that the incidence of mental disorders would likely double in the face of an event like the 2010 earthquake in Haiti [16].

Research on depression and suicide is important for three reasons. First, they represent a large health burden: depression is the leading cause of disability-adjusted life years (DALYs) attributable to mental, neurological, and substance-use disorders in both high-income and low- and middle-income countries (LMICs) [17,18], and suicide accounts for one million deaths per year worldwide [19]. Second, to support the increasing efforts of the WHO and other organizations to implement task-shifting in mental healthcare in low-income settings [20], there is a need to identify optimal referral strategies and key risk factors for screening [21]. Third, known risk factors can be used to design prevention activities to minimize the incidence, morbidity, and mortality associated with depression and suicide in LMICs such as Haiti. Identifying modifiable social risk factors and measuring the impact of poverty, migration, and disasters are among the Grand Challenges in Global Mental Health [18].

While epidemiologic studies of mental disorders are crucial to meeting these goals, the use of instruments that are not culturally-adapted can lead to inaccurate or misleading conclusions; poor data can be more detrimental than lack of data [21]. Therefore, studies should employ culturally-appropriate tools that have undergone rigorous qualitative and ethnographic adaptation, rather than just translation and back-translation [3,20,22-27]. The goal of the present study was to assess the prevalence of and associated factors for depression and suicidal ideation in rural Haiti using a culturally-adapted instrument.

### Depression in Haiti: prevalence and associated factors

Previous studies of depression in Haiti have been limited to specific populations and convenience samples. Moreover, to date, all published studies of depression in Haiti reflect pre-earthquake levels of severity. Among a convenience sample of 258 hospital patients in Haiti, Martsolf (2004) found that 54% met the criteria for major depression using the Center for Epidemiologic Studies – Depression scale [28]. Further research among people living with HIV in Haiti found that eight of the 15 depressive symptoms on the Hopkins Symptom Checklist were endorsed as “quite a bit” or “extremely” by 45-75% of individuals [29]. These psychometric instruments were not validated for use in Kreyòl with Haitian populations, and therefore one cannot confirm that these individuals suffered from clinical depression. Nevertheless, these findings make an important contribution by suggesting a high burden of depressive symptomatology among medically ill Haitians.

In these studies, identified risk factors for depression included: emotional and physical abuse, low socioeconomic status (SES), psychological symptoms of other individuals in the household, and being HIV-positive [29,30]. More research is needed using representative samples to accurately describe the population risk profile of depression in Haiti.

Local cultural and religious factors may interact with conceptualizations of depression, suicidal ideation, and completed suicide in Haiti [31,32]. Idioms of distress that reflect psychosocial complaints among lay persons are often interpreted as other biomedical maladies in healthcare settings (e.g. anemia, gastroesophageal reflux disease) in Haiti [33]. Socio-cultural differences in the understanding of mental distress and suicidal behavior necessitate the study of culturally-specific risk factors and the use of culturally-adapted psychometric screening tools in order to build appropriate prevention and treatment services [21,23,25,28,34]. Yet, the lack of appropriate mental health services and training for health workers may be an even greater challenge than cultural differences for the treatment of mental disorders. For example, lay individuals’ perceptions of biomedical health worker competence addressing mental health issues – rather than differences in cultural explanatory models – may be the dominant determinant of why religious leaders, such as Protestant pastors and Vodou priests, are often preferred over nurses and doctors for psychosocial care in rural Haiti [35].

### Suicide globally and in Haiti

Suicide is a major public health issue, and rates of suicide have increased 60% over the past 45 years, with 85% of suicide deaths occurring in LMICs [19]. There is a dearth of research on suicidal behavior and associated factors in Haiti. Risk factors for suicide documented in other LMICs include: female gender, living in a rural area, young or old age, having low SES, and holding religious beliefs that sanction suicide [36,37]. Also, in India, Taiwan, and China, recent stressful life events, including familial problems and social change [38,39], as well as previous suicide attempts [40,41], have been associated with suicidal behavior. Other studies in Asia have found that young women carry a disproportionate burden of suicide and that there is a weaker association with mental illness than in high-income countries [28,42-47].

Worldwide, there are contrasting findings on the impact of natural disasters, such as earthquakes, on suicide-related behaviors [48-50]. Most research has been conducted in developed countries and suggests that suicide rates decrease following natural disasters [48,49]. However, in one study following Hurricane Katrina in the US, suicidal ideation rates were shown to increase [50].

Globally, only 17% of individuals in low-income countries present to providers in the year prior to suicidal behavior [51]. In Haiti, access to biomedical healthcare is especially low in rural areas. Approximately half of Haitians lack access to primary healthcare, and in rural areas mental health services are virtually nonexistent [52]. Furthermore, of the few psychiatrists practicing in Haiti, most are living and working in urban areas [53], which precludes access for most rural Haitians with depression and/or suicidal ideation.

The present study represents one of the first epidemiologic studies of depression symptomatology, suicidal ideation, and associated risk factors using a culturally-adapted instrument among a representative community sample in Haiti’s Central Plateau. This study aims to inform the development and scale-up of sustainable mental health and psychosocial services in Haiti.

## Methods

### Sample

We conducted a cross-sectional, zone-stratified household survey over six weeks in May-June 2011 in the rural Central Plateau of Haiti. Participants were selected by a stratified design using a modified version of the WHO’s Expanded Program on Immunization “random walk” protocol [54]. Surveys took place in the communal section of La Hoye, with the stratification factor being governmentally-determined zones within the area. Four of the 17 zones were not included in the sampling frame because they had a low population density and were difficult to safely access during the rainy season.

### Research setting

In the study area, livelihood is largely sex-differentiated: males tend to *travay la tè* (work the land), while females *fè k*ò*mes* (do commerce) and care for the family and home [32]. The majority of the population cannot read or write [55]. While everyone speaks Kreyòl, a small minority also speak French (these individuals tend to have higher levels of education) or Spanish, due to the proximity to the Dominican Republic.

Regarding impact of the 2010 earthquake, the Central Plateau sustained little direct damage. However, many areas of the Central Plateau saw significant increases in population due to internally displaced persons. Additionally, many residents of the Central Plateau had family members killed or directly impacted by the earthquake, and many housed displaced family or friends. Despite these devastating and far-reaching impacts, participants made it clear that the earthquake represents one among many challenges that impact mental and physical health in the Central Plateau.

The communal section of La Hoye was chosen as the research site because of its proximity to a non-governmental organization (NGO) that was interested in providing mental health services in the future. The NGO partners with American medical schools and Haitian healthcare personnel to provide year-round medical care in several communal sections in the Central Plateau. While there are three hospitals within a two-hour drive from the research site, the time and resources required to reach these healthcare facilities by foot, horseback, or motorcycle puts them firmly out of reach for the majority of rural Haitians. Instead, they rely on small clinics, many run by NGOs.

At the time of the research, one town in the research setting had a small clinic with one doctor, one nurse, two auxiliary nurses, and one lab technician. Approximately once per week, medical staff conducted mobile clinics, visiting each zone in the communal section once every three months. During the single day of healthcare provision for the zone, dozens of patients would line up, with several often being turned away at the end of the afternoon when rains began. Rather than utilizing formal biomedical care, people could also purchase medicines and herbal remedies at local markets or draw upon a range of traditional supports, including *hougan*-s ^1^This paper utilizes the standard convention of adding –s to indicate plural Kreyol words, rather than –*yo*, the plural indicator in Kreyol. and *manbo*-s (Vodou priests and priestesses), *doktè fey*-s (herbalists), and religious leaders [56,57].

### Instrument

Our survey consisted of demographic information, daily stressors (such as water insecurity), traumatic experiences associated with the earthquake (such as injury), general trauma (such as death of a family member), social support, care seeking behavior, and explanatory models of distress. Demographic information included a SES scale ranging from 0 to 9, representing a count of the number of the following items possessed: tin roof, bicycle, motorcycle, television, radio, cement house, latrine, electricity, and/or a telephone. Assessing household possessions can provide a more objective measure of SES than estimated household income in subsistence economies or when all household members are not included in financial decision making [58,59]. Additional file 1: Table S1 includes a detailed description of all study variables.

Depression symptom burden was assessed using a culturally-adapted Kreyòl version of the Beck Depression Inventory (BDI) [60]. The BDI is a self-report measure of depression symptoms assessed over the past two weeks, with 21 questions answered according to a 0 to 3 severity rating, and an overall range from 0 to 63 [61]. The BDI has been adapted and validated for use in a variety of cultural settings [62-67].

### Local adaptation of tools

The BDI was adapted using a standardized approach for transcultural translation that evaluates semantic, content, construct, and technical equivalence [24,26]. This process involves five steps: translation by bilingual lay individuals, review by bilingual medical professionals working from the local setting, focus group discussions with lay representative members of the target group, back-translation by a bilingual individual blinded to the original instrument, and pilot testing. At each of the five stages, all items are individually assessed for comprehensibility, acceptability, and relevance. These assessments are used to make adjustments to the translation at each stage.

In Haiti, the BDI underwent the process described above using six focus groups comprised of lay men and women [59]. Examples of changes made to the BDI arising from qualitative research include items for “loss of interest” and “indecisiveness.” Loss of interest in the literal translation was interpreted as disinterest in helping others. To capture the construct of anhedonia, the translation was changed to “I am not on it” (*m pa sou sa*), which more closely approximates lack of interest and desire to engage with people and activities. “Indecisiveness” was also changed because the original translation was interpreted as not having the power to decide, rather than not having the concentration and motivation to make decisions. The item was reworded to incorporate a sense of confusion (*pa fouti ka pran yon desisyon*).

The adapted BDI was piloted among a sample of 31 lay participants to assess internal reliability. The Cronbach’s alpha for the pilot sample suggested high reliability (α = 0.89). Of note, the items for sleep difficulties and changes in eating appeared to discriminate poorly between individuals scoring higher or lower on the depression scale, as these items were highly endorsed by nearly all respondents. This phenomenon has been observed in other settings with high levels of gastrointestinal pathology or other physical medical problems, such as rural Nepal [25,62,68,69]. Similarly, medically ill and geriatric populations may have somatic complaints that elevate BDI scores in the absence of clinical depression [63,70]. While these items were not removed, they suggest that total BDI scores may be inflated relative to Western settings. Ultimately, a diagnostic validation study of the Kreyòl BDI is needed to determine the cut-off score with the strongest psychometric properties.

### Survey implementation

One of four trained research assistants administered each survey verbally in Haitian Kreyòl, recording responses on paper. Research assistant training consisted of two days of didactic training on survey delivery, sampling, and basic epidemiologic methods, as well as three days of pilot survey data collection to ensure quality delivery of the BDI. On-going feedback and training took place throughout the period of data collection to ensure appropriate survey implementation. Finalized surveys took approximately one hour to complete.

Four research assistant days were spent in each of the 13 zones, beginning at a central zone location and proceeding in opposite directions, sampling every house encountered. A household was defined as a group of people sleeping in the same *lakou* (compound). Individuals self-identifying as household visitors were ineligible. Because data are not available regarding the precise age structure of the Central Plateau, research assistants rotated asking to speak to a member of a given household aged 18–30, 30–50, or 50+, with individuals under 18 being ineligible. If a person of the preferred age range was not present, the research assistant chose the person closest in age to the preferred age category. Additionally, research assistants alternated recruiting male and female participants. Surveys were double-entered into Excel 2007 [71] each day, and data entry errors were fixed by crosschecking for consistency using Excel Compare v2.4 [72].

### Analysis

Scores on the 21-question culturally-adapted Kreyòl BDI were summed to create an index of increasing symptoms of depression, with potential scores ranging from 0 to 63. Linear scores were employed rather than a categorical cut-point because the Kreyòl BDI has not yet undergone clinical validation. For individuals with missing or no response on BDI questions, we used the individual mean score on completed BDI questions and multiplied this number by 21. A sensitivity analysis showed that using group mean imputation did not meaningfully change associated factors or magnitudes. Suicidal ideation endorsement was defined as responding to BDI item #9 with either: (1) “I have the idea to kill myself but I will not do it,” (2) “I would like to kill myself,” or (3) “I will kill myself if I get the chance”.

We used SAS 9.3 [73] for statistical analyses. Associations were evaluated for statistical significance at α = 0.05 using two-tailed tests. Multivariable linear and logistic models were built, with the outcomes being continuous BDI scores and endorsing suicidal ideation, respectively. Variables considered for inclusion into each model were: age, gender, marital status, education, religion, distance to and type of work, distance to and type of drinking water, care seeking behavior, household size, SES, trauma related to earthquake, general trauma, reported household mental illness, number of children, months without enough food, stigma towards mental illness, explanatory models of distress, alcohol use, care and household help, BDI score minus item 9 (for logistic model only), and endorsing suicidal ideation (for linear model only).

Backward elimination procedures (α = 0.05) were used to arrive at final models. To examine differences between genders, associated factors, and magnitudes, male-only and female-only linear regression models were built in addition to a combined gender model. We *a priori* chose to analyze BDI scores separately by gender because of differences in risk factors for common mental disorders between men and women observed cross-culturally [74-78]. For logistic regression, due to limited events, only a combined gender model was built and variables were screened prior to backward elimination. Only variables with at least one event among individuals endorsing suicidal ideation and a bivariate p <; 0.20 were entered into the logistic model for backward elimination.

To establish significance of individual predictors, t-tests were used for linear regression, and Wald chi-square tests were used for logistic regression. For significance of group predictors (distance to work, education and marital status), chunk F-tests were used for linear regression, whereas likelihood-ratio tests were used for logistic regression. The fit of each final linear model was evaluated using residual diagnostics, partial residual plots, Cook’s distance, leverage values, and variance inflation factors. Hosmer and Lemeshow’s goodness-of-fit test and the area under the receiver operating characteristic (ROC) curve were used to evaluate fit for the logistic model.

### Ethical approval

This project was approved by the Institutional Review Board of Emory University (expedited review, #IRB00042396) and the Haitian Ministry of Health. Prior to asking survey questions, research assistants completed an informed consent process with each participant in Kreyòl. Because the majority of rural Haitians are not literate, verbal consent was used. Regarding management of high risk participants in the study, an American licensed clinical social worker from our research team conducted a follow-up diagnostic interview with anyone responding with a 2 or 3 on the adapted BDI suicidal ideation item to assess level of distress and refer those who endorsed active suicidal ideation.

## Results

Overall, survey response rate was high: of 551 households visited, 98% of individuals agreed to participate and completed the survey. Of 540 surveys completed, 132 were conducted during research assistant training to ensure proper delivery of psychometric screening tools, leaving 408 eligible for final analysis. Over 89% of our sample answered all BDI questions, with 8.1% missing one question and 2.5% missing more than one question. The 43 individuals with missing BDI data did not have significantly different age or gender distributions compared to the sample as a whole. Additional file 2: Figure S1 depicts the distribution of BDI scores.

Basic demographics showed our sample to be 49.5% female and 50.5% male, with a median age of 39 (see Table 1). Over 57% had never attended school, 96.8% did not have enough food for one or more months a year, and 62.8% responded that a member of their household suffered from stress, sad heart (*kè pa kontan)*, or sadness that made daily life difficult.

**Table 1:** Demographic and behavioral characteristics of 408 adults completing household survey in the rural Central Plateau of Haiti, May – June, 2011

**Demographic or behavioral characteristic**	**n (%) unless noted**	**IQR**
**Imputed Beck Depression Inventory Score**	18 (median)	15
**Age**	39 (median)	22
Missing	1 (0.25)	
**Gender**		
Female	202 (49.5)	
Male	206 (50.5)	
**Relationship status**		
Single	65 (15.9)	
Cohabiting (not married)	156 (38.2)	
Married	146 (35.8)	
Divorced	9 (2.2)	
Widowed	31 (7.6)	
Missing	1 (0.25)	
**Education**		
No school	233 (57.1)	
Some primary school	59 (14.5)	
Finished primary school	89 (21.8)	
More than primary school	25 (6.1)	
Missing	2 (0.49)	
**Distance to work**		
Travels over 1 hour to work	72 (17.7)	
Travels 1 hour or less to work	226 (55.4)	
Does not work	109 (26.7)	
Missing	1 (0.25)	
**Months with not enough food**		
Always enough food	13 (3.2)	
1 to 3 months	183 (45.0)	
3 to 6 months	99 (24.3)	
6 to 9 months	94 (23.1)	
9 to 12 months	18 (4.4)	
Missing	1 (0.25)	
**Endorses current suicidal ideation**	25 (6.1)	
**Travels over 1 hour to doctor**	231 (56.6)	
Missing	5 (1.2)	
**Someone in household suffers from stress/sad heart/sadness that makes life difficult**	256 (62.8)	
Missing	3 (0.74)	
**Ever had life-threatening illness**	159 (39.0)	
**Had death in family (not exclusively due to earthquake)**	248 (60.8)	
**Had family who died in the earthquake**	188 (46.1)	
**Had more people come live in house after earthquake**	238 (58.3)	
**Has someone who can help care for them when sick**	81 (19.9)	
Missing	1 (0.25)	
**Answered that spirits cause stress/sad heart/sadness**	39 (9.6)	
**Answered that disasters can cause stress/sad heart/sadness**	252 (61.8)	
**Use alcohol**	78 (19.1)	
Missing	2 (0.49)	
**Ever visited a Vodou priest**	50 (12.3)	

### Prevalence of depression symptomatology and current suicidal ideation

The mean score on the imputed BDI was 20.4 (CI: 19.3-21.5; median: 18), with 41.7% scoring 20 or greater and 22.7% scoring 29 or greater. Of the 408 participants, 6.13% (N = 25) endorsed current suicidal ideation, and all endorsing active suicidal ideation were confirmed as suicidal through follow-up diagnostic interviews.

### Fit of final multivariable models

For the combined, male-only, and female-only linear models, each final model revealed no significant lack of fit or multicollinearity and achieved R-squared values of 0.40, 0.49, and 0.34, respectively. For the final logistic model, Hosmer and Lemeshow’s goodness-of-fit test revealed no significant lack of fit (p = 0.75), and the model achieved excellent discrimination, with the area under the ROC curve being 0.86. Internal consistency of the locally-adapted BDI was high, achieving a Cronbach’s alpha of 0.86.

### Factors associated with BDI scores for both males and females

Older age was associated with increased BDI scores for both genders. Each 10-year increase in age was associated with an increase of 1.1 BDI points for men (p = .009) and 1.8 points for women (p = .002); (see Table 2). Women, on average, scored 2.1 points higher than men on the BDI (p = .031). In the combined gender model, traveling over one hour to a doctor was associated with scoring 2.4 points higher on the BDI (p = .012). However, distance to formal healthcare services was not significantly associated with BDI scores in male-only or female-only models. Endorsing suicidal ideation was associated with an increase of 14 points on the BDI for men (p <; .001) and 7.7 points for women (p = .009). Collapsing across genders, having a death in one’s family and having had a life-threatening illness were associated with a 2.7 point (p = .013) and 2.6 point (p = .006) increase in BDI scores, respectively.

**Table 2:** Multivariable linear regression of combined, male-only, and female-only models for individuals completing household survey in the rural Central Plateau of Haiti May – June 2011, with scores on locally-adapted Beck Depression Inventory as linear outcome

**Covariates**	**Combined**	**Males only**	**Females only**
	**(N = 395**^**a**^**)**	**(N = 205**^**b**^**)**	**(N = 198**^**c**^**)**
	**aβ (95% CI)**^**d**^	**aβ (95% CI)**^**d**^	**aβ (95% CI)**^**d**^
Age	0.14 (0.06, 0.22)^†^	0.11 (.030, 0.20)*	0.18 (0.07, 0.29)*
Female gender	2.1 (0.18, 4.0)*	n/a	n/a
SES scale	n.s.	n.s.	−1.5 (−2.6, -0.39)*
Endorsed suicidal ideation	11.1 (7.3, 14.9)^†^	14.0 (9.2, 18.9)^†^	7.7 (1.9, 13.4)*
Travels over 1 hour to doctor	2.4 (0.52, 4.2)*	n.s.	n.s.
Last time sick went to hospital	n.s.	−5.0 (−8.6, -1.4)*	n.s.
Months without enough food (3 month intervals)	1.1 (0.04, 2.1)*	2.0 (0.57, 3.4)*	n.s.
Had death in family	2.7 (0.57, 4.9)*	3.8 (1.0, 6.6)*	3.8 (0.72, 6.9)*
Ever had life-threatening illness	2.6 (0.77, 4.5)*	3.1 (0.67, 5.6)*	3.6 (0.74, 6.5)*
Someone in household has stress / sad heart / sadness	2.6 (0.58, 4.7)*	n.s.	4.1 (1.1, 7.0)*
Had more people come live in their house after earthquake	2.6 (0.74, 4.5)*	3.8 (1.4, 6.2)*	n.s.
Family member died in earthquake	n.s.	n.s.	3.5 (0.59, 6.4)*
Spirits cause stress, sadness	4.9 (1.6, 8.1)*	n.s.	5.9 (1.5, 10.3)*
Disasters cause stress, sadness	4.8 (2.8, 6.8)^†^	7.8 (5.2, 10.4)^†^	n.s.
**Distance to work**^**e**^			
Travels over 1 hr to work	8.0 (5.1, 10.8)^†^	12.2 (8.3, 16.1)^†^	6.8 (2.7, 11.0)^†^
Travels 1 hr or less to work	5.9 (3.5, 8.2)^†^	7.8 (5.2, 10.4)^†^	3.3 (0.02, 6.5)*
Does not work	(reference)	(reference)	(reference)
**Education**^**f**^			
More than primary school	3.2 (−0.94, 7.3)	n.s	7.7 (2.0, 13.4)*
Finished primary school	2.9 (0.37, 5.5)*	n.s	4.8 (0.73, 8.8)*
Some primary school	3.1 (0.31, 5.9)*	n.s	4.7 (0.19, 9.1)*
No formal schooling	(reference)	n.s	(reference)
**Marital Status**^**g**^			
Widowed	2.0 (−2.6, 6.6)	n.s	n.s
Divorced	7.5 (0.70, 14.4)*	n.s	n.s
Cohabitating	−1.1 (−4.1, 1.8)	n.s	n.s
Married	−3.0 (−6.2, 0.09)	n.s	n.s
Single	(reference)	n.s	n.s

*P <; .05 *(T*_*1*_*).*

^†^ P <; .001*(T*_*1*_*).*

n.s. = Eliminated through backward selection for given model.

^**a**^ Of 408 total respondents, excludes 1 with missing data for education, 1 with missing data for distance to work, 3 with missing data for household mental illness, 1 with missing data for months without food, 5 with missing data for time to doctor, 1 with missing data for age, and 1 with missing data for education and marital status.

^**b**^ Of 206 total males, excludes 1 with missing data for distance to work.

^**c**^ Of 202 total females, excludes 2 with missing data for education, 1 with missing data for household mental illness, and 1 with missing data for age.

^**d**^ Variables considered for inclusion: age, gender, marital status, education, religion, distance to and type of work, distance to and type of water, care seeking behavior, household size, SES, trauma related to earthquake, general trauma, reported household mental illness, number of children, months without enough food, stigma towards mental illness, explanatory models of distress, alcohol use, care and household help, opinions on mental illness causation, and endorsing suicidal ideation.

^**e**^ Construct p-values for distance to work were p <; .001 (combined), p <; .001 (males only), and p = .005 (females only).

^**f**^ Construct p-values for education were p = .035 (combined), non-significant (males only), and p = .012 (females only).

^**g**^ Construct p-values for marital status were p = .002 (combined), non-significant (males only), non-significant (females-only).

Distance to work was strongly associated with BDI scores for both genders (p <; .0001 men; p = .005 women). Compared to those who do not work, traveling over one hour to work was associated with a 12.2 point increase on the BDI (p <; .001) for men and a 6.8 point increase for women (p = .002).

Marital status was associated with BDI scores only in the model collapsed across genders (p = .002). Compared to those who are single, being divorced was associated with a 7.5 point increase on the BDI (p = .031).

### Factors associated with BDI scores for males only

Among men only, responding that the last time they were sick they went to the hospital was a protective factor, associated with scoring 5.0 points lower on the BDI (p = .007). Each three-month increment that men stated they did not have enough food for their family was associated with scoring 2.0 points higher on the BDI (p = .006). Having more people come live in one’s house after the earthquake was associated with men scoring 3.8 points higher on the BDI (p = .002). Stating that disasters are the primary cause of stress, sad heart, or sadness was associated with men scoring 7.8 points higher on the BDI (p <; .001).

### Factors associated with BDI scores for females only

For women only, each additional item possessed on the SES scale was associated with scoring 1.5 points lower on the BDI (p = .008). Reporting that someone in their house suffers from stress, sad heart, or sadness, that a family member died in the 2010 earthquake, or that spirits can cause stress, sad heart, or sadness was associated with scoring 4.1 (p = .008), 3.5 (p = .019) and 5.9 (p = .009) points higher on the BDI, respectively. Level of education was significantly associated with BDI scores for women only (p = .012). Compared to those with no formal education, having more than a primary school education was associated with scoring 7.7 points higher on the BDI (p = .008).

### Factors associated with suicidal ideation

Score on the BDI (minus item #9) was the factor most strongly associated with suicidal ideation. For each ten point increase on the BDI, the odds of endorsing suicidal ideation increased 2.5 fold (p <; .0001; see Table 3). Individuals who use alcohol were 3.3 times as likely to endorse suicidal ideation (p = .012). Individuals who reported ever having gone to a Vodou priest were 3.2 times as likely to endorse suicidal ideation (p = .037). Last, individuals reporting that they did not have someone to care for them if sick were 5.5 times as likely to endorse suicidal ideation (p = .043).

**Table 3:** **Multivariable logistic regression of 405**^**a**^**individuals completing household survey in the rural Central Plateau of Haiti, May-June, 2011, using current suicidal ideation endorsement on Beck Depression Inventory (BDI) as binary outcome**

**Covariates**	**aOR (95% CI)**^**b**^
Imputed BDI score, excluding item #9 (10 point increase)	2.5 (1.7, 3.6)^†^
Does not have someone to care for them if sick	5.5 (1.1, 28.6)*
Use alcohol	3.3 (1.3, 8.2)*
Ever been to a Vodou priest	3.2 (1.1, 9.5)*

*P <; .05 (Wald ÎÂ§^2^).

^†^ P <; .001 (Wald ÎÂ§^2^).

^**a**^ From 408 total respondents, excludes 2 with missing data on alcohol use, and 1 with missing data on whether they have care if sick.

^**b**^ Variables considered for inclusion: age, gender, marital status, education, religion, distance to and type of work, distance to and type of water, care seeking behavior, household size, SES, trauma related to earthquake, general trauma, reported household mental illness, number of children, months without enough food, stigma towards mental illness, explanatory models of distress, alcohol use, care and household help, and BDI score minus suicidal question (item #9).

## Discussion

In a rural population of adults in Haiti’s Central Plateau, over 6% of the population endorsed current suicidal ideation, and the mean level of depression symptomatology was a score of 20.4 on the BDI. Factors significantly associated with depression symptom burden were: age, female gender, SES, recent life stressors such as life-threatening illness or death in the family, daily life stressors such as lack of food or traveling long distances to work, explanatory models of distress, endorsing current suicidal ideation, marital status, and education level. Current suicidal ideation was most strongly associated with scores on our culturally-adapted KreyÃÂ²l BDI, followed by alcohol use, having been to a Vodou priest, and lacking care if sick.

The observed associations of depression symptom burden with age, female gender, distance from healthcare, and exposure to stressful life events, including disaster-related stressors, are consistent with literature in other LMICs and post-disaster settings [79-81]. Additionally, the associations of depressive symptomatology with SES and psychological symptoms of household members are consistent with previous findings among medically-ill samples in Haiti [29,30]. Social exclusion might explain the 7.5 point increase on the BDI for individuals who have been divorced; in high-income countries, divorce is one of the major life events precipitating depression [82]. Furthermore, the rarity of divorce in Haiti may also contribute to its stigma and subsequent social alienation. Factors related to healthcare were also associated with depression symptom burden; traveling more than one hour to see a doctor and suffering from a major illness were both associated with higher BDI scores.

Explanatory models, i.e. the perceived causes of mental distress [83,84], were also associated with BDI scores. In our sample, over 60% of participants identified disasters as potentially causing sadness or stress, while 10% endorsed that spirits could cause mental distress. Among women, the belief that spirits cause sadness was associated with higher BDI scores. Among men, the belief that disasters cause sadness was associated with higher BDI scores. In some literature, holding an external locus of control, characterized by a belief that factors outside the self primarily drive wellbeing, is associated with greater psychiatric distress [85-88]. In our sample, the nature and cause of the observed gender difference is unclear.

In recent priority setting regarding major mental health research questions in humanitarian settings, such as natural disasters, the top rated issue was the identification of major stressors for populations in complex emergencies [89]. Although the study area did not directly experience damage from the 2010 earthquake, significant associations were found between depression and earthquake-related variables. Among men, having individuals move into one’s house following the earthquake was associated with poorer mental health outcomes. Among women, having a relative die in the earthquake was associated with greater symptoms of depression. Thus, for men, structural and economic consequences of the earthquake were associated with symptoms of depression, whereas for women, emotional consequences may have been more significant. Taken together, these findings suggest that psychosocial and other mental health responses to disasters may be shortsighted if only focused on areas directly affected. Additionally, interventions to reduce mental distress associated with disasters should consider unique needs based on gender.

Two surprising findings were the associations of education among women and employment among both genders with depression symptomatology. Women with more than a primary-level education scored 7.7 points higher on the BDI compared to uneducated women, even when controlling for age and other potential confounders. This may be due to the socioeconomic context of the rural, agriculturally-focused study region from which educated individuals are more likely to migrate to urban areas to escape low-paying agricultural or household work. The expectation may be that educated women will leave the region to gain employment in Port-au-Prince or abroad. Accordingly, women who are educated but do not leave may be seen as failing to reach their potential, and these women may suffer dissonance between the model of life learned about through education and their current circumstances. In Afghanistan, education, hope, and aspirations have negative associations with mental health in some groups, and this has been described as a disconnect between what is expected and what is possible in that environment [90]. A similar disconnect between aspirations and reality may help explain the association between employment and poorer depression outcomes for both genders. Those employed in rural Haiti may primarily be unskilled laborers traveling away from home for low-paying employment. In-depth qualitative research is required to further elucidate these associations in Haiti.

It is not possible to make claims about prevalence of clinical depression in this study because the Kreyol BDI has not been clinically validated. However, it is worth noting that 41.7% of our respondents scored 20 or greater on our adapted BDI, which is the cut-off for moderate depression in American samples [61]. This is comparable to a study in rural Nepal conducted after the ceasefire concluding the decade-long civil war; that study found a depression prevalence of 40.6% using a validated Nepali BDI with a cutoff of 20 or higher for clinical depression [91]. The Haitian and Nepali populations share a history of poverty, lack of medical care, and structural violence, in addition to recent disasters. The putative rate of 41.7% depression in this Haitian sample is also comparable to depression rates observed in war-affected Liberia (prevalence 40%) [92] and Uganda (prevalence 44.5%) [93], as well as among Burmese refugees in Thailand (prevalence 40.8%) [94]. This suggests that the combination of man-made or natural disasters against a backdrop of poverty, poor healthcare, and other structural violence factors is associated with widespread prevalence of depression symptomatology.

The association between suicidal ideation and depression has been demonstrated repeatedly in cross-national WHO studies of suicide-related behaviors [36]. However, as pointed out by the WHO studies, suicidal ideation does not consistently predict suicide attempts. The WHO estimates that the *lifetime* cross-national prevalence rate of suicidal ideation is 9% [36], which is greater than the 6% current prevalence in rural Haiti. A limitation of this comparison is that the WHO study did not report rates of current suicidal ideation. One concern, based on findings of patients of Black Caribbean ancestry in the United Kingdom, is that this population may have a higher ratio of attempts to ideation compared with White European populations [95]. Future research should investigate whether this is in fact the case in Haiti.

The association of alcohol use and suicidal ideation is not surprising; this association has been identified in numerous studies [96-100]. Substance abuse not only predicts suicidal ideation, but is a strong predictor of suicide attempts and completions, in part because it can increase impulsivity [101]. Lack of someone to care for the respondent when sick also predicted suicidal ideation. Loneliness and lack of social support, as with substance abuse, are common risk factors in most studies of suicidality [102-105]. The association with prior visits to a Vodou priest is consistent with the finding that persons in low-income countries who have attempted suicide may have visited a non-psychiatric health worker, such as a traditional healer, prior to attempting suicide [51].

There is a paucity of accurate demographic data for the Central Plateau. Despite this, if we assume our sample is generally representative, we would expect 17,460 individuals to endorse current suicidal ideation in rural areas of the Haitian Central Plateau given 2009 population estimates for persons over 18 years of age (284,948) and our estimated population rate of current suicidal ideation [106,107].

### Implications for referral, treatment, and prevention

The goal of this study was to elucidate patterns of burden for depression symptomatology and suicidal ideation in rural Haiti in order to identify potential actions for referral, treatment, and prevention. Regarding referral, having visited a Vodou priest was associated with suicidal ideation, whereas having visited a biomedical provider was not. This suggests that persons with suicidal ideation are likely to have visited a Vodou practitioner. Therefore, referrals from Vodou priests and other community and mental health resources may be ideal to support those with current thoughts of ending their life. That said, partnerships with traditional healers can have negative outcomes as well, such as being more expensive than biomedical treatment [108]. Religious and traditional healers may also worsen stigma based on the explanatory models they invoke [109,110]. These risks should be taken into account when developing referral systems in Haiti.

Our research indicates that natural disasters, such as earthquakes, can have far reaching psychological effects, including on individuals not directly in the earthquake-affected region. Therefore, depression screening programs should attempt to reach those who are indirectly affected, such as those accepting displaced persons and those with relatives who died in the disaster. For example, individuals within households that hosted displaced persons could all undergo screening, as hosts are also at risk of increased symptoms of depression. An additional screening group could be those who have suffered recent losses such as widows and those with a death in the family. Individuals who deal with these groups, such as religious leaders who assist in funerary rites, could be a source of referrals. Rather than medical professionals, religious leaders may be better positioned and able to distinguish between culturally-appropriate grief expressions versus more pathological and impairing psychiatric sequelae.

For treatment, while psychiatric services need to be improved in general, there are specific populations who would particularly benefit from improved mental healthcare. One such group is substance abusers who are at risk of suicidal ideation and likely suicide attempts. Packages of care for substance abuse are available, and trials have been conducted in a range of LMICs [111]. These could be piloted and optimized for Haiti’s Central Plateau. Cross-cultural studies of substance abuse treatment have shown that involvement of the family is extremely important, and this should be emphasized in Haiti. Another important treatment group is the medically ill. In our study, persons with medical problems were at increased risk for depression symptoms, a finding that is consistent with global literature on medical illness and mental health [112]. Therefore, interventions that target depression, anxiety, and demoralization among the medically ill could significantly lower the burden of mental illness [113-115], while also improving medical outcomes [112].

The finding that depression symptom burden was higher among women may be associated with reproductive life events [77]. In Pakistan, community-based psychotherapeutic programs have successfully reduced the burden of maternal depression [116]. Similar interventions would be feasible in Haiti given the low level of technical psychiatric knowledge needed for implementing such a program [117]. Further research is needed to elucidate gender-specific causes and effects of depression in Haiti.

For prevention of mental illness, structural interventions to reduce poverty are likely indispensible. Poverty and mental disorders easily become vicious cycles; therefore, reducing poverty will help prevent mental disorders and reduce barriers to recovery among those who already suffer from psychological distress [118,119]. Food security is one component of poverty reduction that has shown a strong association in this population and thus should be targeted. In addition, improving the quality and availability of medical care also will likely prevent some of the current burden of depression. This medical care should include not only clinical services, but also community outreach for persons with current medical conditions who may need home health assistance. The observed relationship between reported lack of a caregiver when sick and suicidal ideation points toward the need for community-based psychosocial support to reach out to ill persons lacking family support.

Ultimately, there are a number of current models for improving mental healthcare in low-income settings [21,120,121]. An additional key will be to address the policy barriers to implementing these evidence-based practices in Haiti.

### Strengths and limitations

Our study has several limitations. First, compared to the 13 zones sampled, the four zones excluded due to inaccessibility could have higher depression symptomatology and suicidal ideation due to further isolation from biomedical, mental health, and psychosocial services. Second, due to lack of an up-to-date census or list of households, we had to rely on the WHO’s “random walk” protocol. Nevertheless, the “random walk” protocol has been shown to give accurate and precise results even when compared to a randomized probability sample based on a household census [122]. Third, since our BDI instrument has not been clinically validated, we cannot establish specific cut-offs to determine the absolute prevalence of major depression. Regardless, the use of a culturally-adapted BDI is a major strength of our study and aids in ensuring valid determination of risk factors, even if absolute prevalence cannot be estimated [123]. An additional strength is that all reported cases of active suicidal ideation were confirmed through follow up by a licensed clinical social worker.

## Conclusion

We found high depression scores in the Central Plateau of Haiti using a culturally-adapted BDI screening tool. Our data suggest the need for mental health services to reach specific groups at higher risk for serious mental disorders or suicidal ideation, while also highlighting the possibility of screening for critical life events. Screening could be conducted in biomedical, religious, and Vodou healing contexts. For prevention, poverty reduction and improved healthcare access are key elements. For treatment, general psychiatric services, psychosocial services for the medically ill and their families, and substance abuse interventions should be explored. Paradoxical associations related to education and employment require further exploration.

In conclusion, our findings show a high burden of depression symptomatology and suicidal ideation in rural Haiti and can guide the development and scaling-up of screening, intervention, and prevention activities. Additional studies are urgently needed to characterize the burden and associated factors of common mental illness in the general population of Haiti.

## Endnote

^a^ This paper utilizes the standard convention of adding –s to indicate plural Kreyol words, rather than –*yo*, the plural indicator in Kreyol.

## Competing interests

The authors declare that they have no competing interests.

## Author’s contributions

BW, AH, BNK, and BAK devised the survey instrument. BNK provided locally-adapted screening tools for survey. BW and AH implemented the survey in Haiti with supervision from BAK. KEM participated in the design and implementation of the study. BW conceived and carried out data analysis independently, with supervision from BAK. BW drafted the first version of the manuscript. All authors read and approved the final manuscript.

## Footnotes

^1^This paper utilizes the standard convention of adding –s to indicate plural Kreyol words, rather than –*yo*, the plural indicator in Kreyol.

## Pre-publication history

The pre-publication history for this paper can be accessed here:

http://www.biomedcentral.com/1471-244X/12/149/prepub

## Supplementary Material

Additional file 1 **Table S1.** Detailed list of variables considered for inclusion into each multivariable model. Click here for file

Additional file 2 **Figure S1.** Scores on imputed 21-question locally-adapted Beck Depression Inventory (BDI) for 408 individuals living in the rural Central Plateau of Haiti who completed household survey, May-June, 2011. Click here for file
