# Supplementary material for: Depression, suicidal ideation, and associated factors: a cross-sectional study in rural Haiti
Source: BMC Psychiatry. 2012 Sep 19;12:149. doi: 10.1186/1471-244X-12-149 (PMC3515455; doi:10.1186/1471-244X-12-149)

**Supplementary Table 1. Detailed list of variables considered for inclusion into each multivariable model.**

| **Variable** | **Question** | **Question format** | **Answer Choices** |
| --- | --- | --- | --- |
| Age | What age are you? | Write-in | N/A |
| Gender | Are you a woman or a man? | Circle one | Female, male |
| Marital status | What is your marital status? | Multiple choice | Single, cohabiting, divorced, widowed, married |
| Education | How much education do you have? | Multiple choice | No formal schooling, some primary, finished primary, some high school, finished high school, finished graduate/professional |
| Religion | What is your religion? | Multiple choice (choose as many as apply) | Catholic, Protestant, Baptist, Episcopal, Vodou, no religion, other (write-in) |
| Distance to work | How much time does it take to arrive at your work? | Multiple choice | Do not work, 0-15 minutes, 15-30 minutes, 30-60 minutes, 1-2 hours, more than 2 hours |
| Type of work | What work do you do? | Multiple choice (choose as many as apply) | Do not work, commerce, teacher or professor, taxi driver, seamstress, farmer, raise animals, other (write-in) |
| Distance to drinking water | How much time does it take to get drinking water and return to your house? | Multiple choice | 0-15 minutes, 15-30 minutes, 30-60 minutes, 1-2 hours, more than 2 hours |
| Type of drinking water | Where do you get your water for drinking? | Multiple choice | River, well, spring, covered well, pipe/tap, other (write-in) |
| Care seeking behavior | The last time you were sick where did you go for help? | Multiple choice | Family, friends, Vodou priest, Vodou priestess, church pastor or priest, hospital or clinic, community health worker, NGO, chief of community, herbal healer, did not go anywhere, other (write-in) |
| Care seeking behavior | How much time does it take for you to get to a hospital or clinic? | Multiple choice | Never go to doctor, 0-15 minutes, 15-30 minutes, 30-60 minutes, 1-2 hours, more than 2 hours |
| Care seeking behavior | Have you ever been to a Vodou priest for help (treatment)? | Binary | Yes/no |
| Care seeking behavior | Have you ever been to an herbal healer for help (treatment)? | Binary | Yes/no |
| Care seeking behavior | Have you ever been to a hospital for help (treatment)? | Binary | Yes/no |
| Care seeking behavior | Have you ever been to a church priest or pastor for help (treatment)? | Binary | Yes/no |
| Household size | How many people total (adults and children) live in this household (Kreyol: *Lakou*) | Write-in | N/A |
| SES scale | Do you have? | Multiple choice (choose as many as apply) | Tin roof, bicycle, motorcycle, television, radio, cement house, latrine, electricity, telephone |
| Trauma related to earthquake | Which of the following did you experience regarding the earthquake? | Multiple choice (choose as many as apply) | I was in a town hit by the earthquake, I was injured/hurt, a family member of mine was killed, new people moved into my house |
| General trauma | Which of the following have you *ever* experienced? | Multiple choice (choose as many as apply) | Been attacked with a weapon (gun, knife, machete), had possessions stolen, been in a machine (car, motorcycle) accident, experienced large-scale violence (war, violent protest), been affected by a large fire, had a large flood in house or farm, had someone die in my family, had a life-threatening illness, have been raped |
| Reported household mental illness | Does anyone in your household suffer from sadness, unhappy heart, or stress that makes life difficult? | Binary | Yes/ no |
| Number of children | How many children live in this household (Kreyol: *Lakou*) | Write-in | N/A |
| Months with not enough food | How many months of the year do you not have enough food | Multiple choice | Always enough food  1 to 3 months  3 to 6 months  6 to 9 months  9 to 12 months |
| Stigma towards mental illness | If someone suffers from sadness, unhappy heart, or stress that makes life difficult, is it their fault? | Multiple choice | Never, sometimes, always |
| Explanatory models of distress | What is capable of causing sadness, unhappy heart, or stress that makes like difficult? | Multiple choice (choose as many as apply) | Spirits, alcohol or drugs, thinking too much, problems with family or community members, disasters, bad luck, lack of work, other (write-in) |
| Alcohol use | Do you drink alcohol? | Binary | Yes/no |
| Care and household help | Do you have someone who can… | Binary (yes/no) | Care for you if sick, go to the market or buy food for you, loan you money if you need it (not from bank), make food for you, give you advice |
| BDI score | Contact authors for full list of locally-adapted Beck Depression Inventory questions in Kreyol | Multiple choice | Questions 1-21 |
| Endorsed current suicidal ideation (BDI item # 9) | In the past 2 weeks - thoughts of killing myself | Multiple choice | I have never thought of killing myself  I have thought of killing myself but I will not do it  I would like to kill myself  I will kill myself if I get the chance |


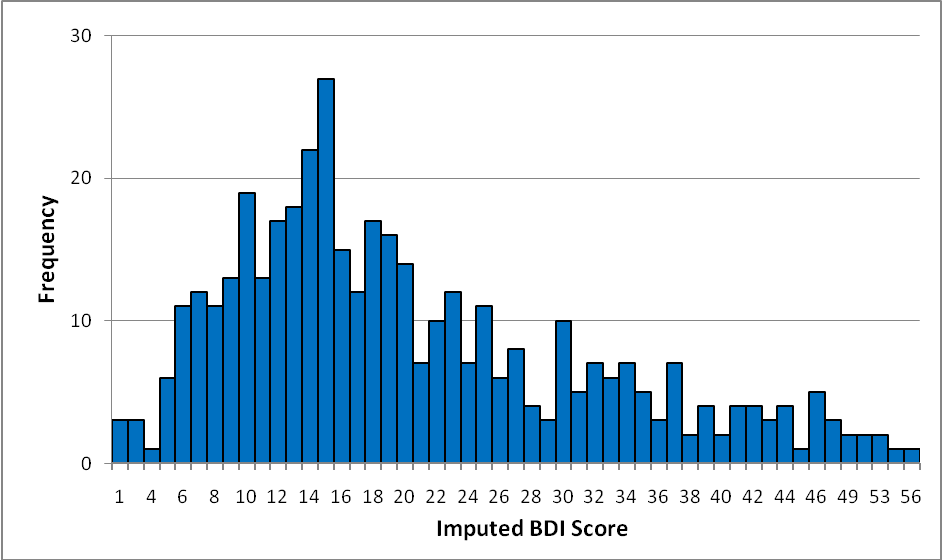

Supplement: Additional file 1 — Table S1. Detailed list of variables considered for inclusion into each multivariable model. [file 1471-244X-12-149-S1.doc]
