# Supplementary material for: Depression, suicidal ideation, and associated factors: a cross-sectional study in rural Haiti
Source: BMC Psychiatry. 2012 Sep 19;12:149. doi: 10.1186/1471-244X-12-149 (PMC3515455; doi:10.1186/1471-244X-12-149)
Supplement: Additional file 2 — Figure S1. Scores on imputed 21-question locally-adapted Beck Depression Inventory (BDI) for 408 individuals living in the rural Central Plateau of Haiti who completed household survey, May-June, 2011. [file 1471-244X-12-149-S2.doc]

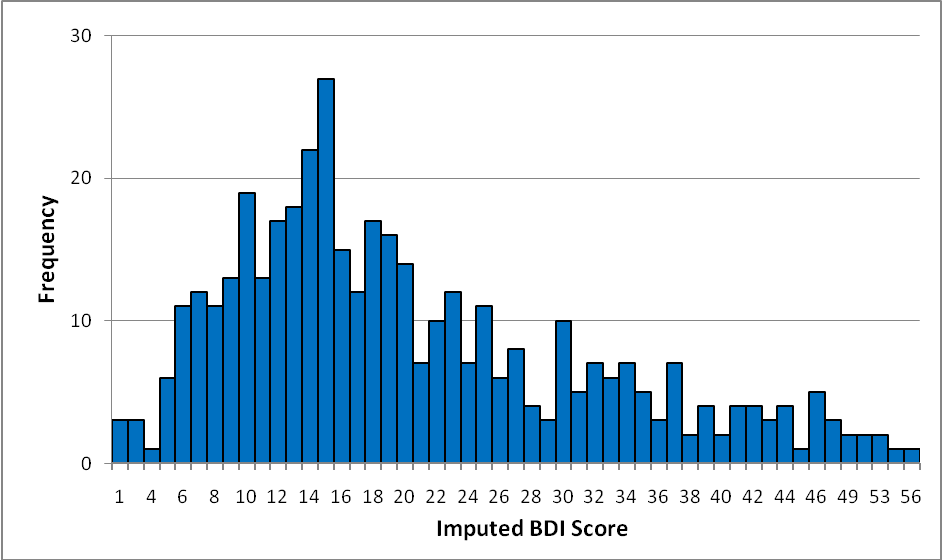


**Supplementary Figure 1** - **Scores on imputed 21-question locally-adapted Beck Depression Inventory (BDI) for 408 individuals living in the rural Central Plateau of Haiti who completed household survey, May-June, 2011.**
